# Embodying Deficiency Through ‘Affective Practice’: Shame, Relationality, and the Lived Experience of Social Class and Gender in Higher Education

**DOI:** 10.1177/0038038515589301

**Published:** 2015-06-30

**Authors:** Vik Loveday

**Affiliations:** Goldsmiths, University of London, UK

**Keywords:** affect, deficiency, embodiment, gender, practice, social class, shame, value

## Abstract

Based on empirical research with participants from working-class backgrounds studying and working in higher education in England, this article examines the lived experience of shame. Building on a feminist Bourdieusian approach to social class analysis, the article contends that ‘struggles for value’ within the field of higher education precipitate classed judgements, which have the potential to generate shame. Through an examination of the ‘affective practice’ of judgement, the article explores the contingencies that precipitate shame and the embodiment of deficiency. The article links the classed and gendered dimensions of shame with valuation, arguing that the fundamental relationality of social class and gender is not only generative of shame, but that shame helps in turn to structure both working-class experience and a view of the working classes as ‘deficient’.

## Introduction: Situating Social Class, Value and ‘Affective Practice’ in Higher Education

The article begins from the premise that social class is intimately tied to processes of valuation that operate through ‘devices of distancing and distinction’ (Skeggs and Loveday, 2012). It explores the lived experience of shame by looking at the ‘affective practice’ (Wetherell, 2012, 2014) of judgement – a key ‘device’ in determining ‘person-value’ (Skeggs, 2010a, 2011) or ‘person-deficit’. By paying attention to the question of how ‘social formations grab people’ (Wetherell, 2012: 2), the article seeks to contextualise the profoundly social nature of shame (Chase and Walker, 2013) and the classed and gendered conditions that coalesce in its production.

The context of the article is higher education (HE) in England, an interesting field for research on classed relations given that ‘[t]he size of the socio-economic gap in participation really is substantial’ (Vignoles, 2013: 115). There has been a considerable expansion in the HE sector since the 1960s (see Boliver, 2011) and Widening Participation initiatives have more recently sought to address further inequitable access to universities.^1^ Working-class participation in HE is represented in policy discourses (see e.g. Milburn, 2012) as an instrument in the facilitation of social mobility for those working-class people who are able to participate (see also Loveday, 2014b). Yet Boliver (2011: 240) claims that notwithstanding this expansion in access, ‘social class inequalities in British higher education have been both maximally and effectively maintained.’ The Office for Fair Access reported recently that ‘the most advantaged 20 per cent of young people were 2.5 times more likely to go to higher education ... than the most disadvantaged 40 per cent’ (OFFA, 2014: 2), and social class background also affects the type of institution attended^2^ (Reay et al., 2009), retention (Quinn et al., 2005) and overall outcome (Stuart et al., 2012).

In this article, I take a feminist Bourdieusian approach to social class analysis in order to explore one particular dimension of the lived experience of staff and students from working-class backgrounds in English higher education institutions (HEIs): shame. Skeggs (2010a: 339) notes that: ‘When we examine the historical production of the concept class what we see is how it has operated to conceptualise inequalities of different kinds, of which identity is only one aspect.’ I am interested here in the subjective experience of class (as opposed to objective ‘measures’ of social class location), and the meaning actors attribute to their experiences. Through the conceptual troika of habitus, field and capital (economic, cultural, social and symbolic), Bourdieu provides a dynamic and relational framework for thinking through how multiple forms of advantage are reproduced by some groups *at the expense of others*, that is, through practices of exclusion (see also Toscano and Woodcock, forthcoming). Bourdieu (2000: 173) notes:

Because dispositions are the product of the incorporation of objective structures and because expectations always tend to adjust themselves to chances, the instituted order always tends to appear, even to the most disadvantaged, if not as self-evident, natural, at least as more necessary, more self-evident than might be thought.

The use of the concept of habitus – as ‘embodied history’ (Bourdieu, 1990a: 56) acquired through practice – facilitates an analysis of how relations between actors within delimited fields of action, as well as the relationship of actors to those fields, may forestall or foreclose capital accumulation under the guise of the ‘natural’.

While Bourdieu has been critiqued for failing to develop fully the role of gender in his work, his concepts have been ‘appropriated’ (Moi, 1991) by feminist thinkers concerned to examine the intersection of class with other processes, such as gender and ‘race’.^3^ Skeggs’s notion of ‘person-value’ (Skeggs, 2011) is particularly instructive in exploring how ‘systems of inscription, exchange, valuing, institutionalization and perspective provide the conditions of possibility for being read by others in the relationships that are formed between groups’ (Skeggs, 2004a: 2). For example, the inscription of lack – and the naturalisation of deficiency– in the representation of working-class lives and experience is well-documented (Charlesworth, 2000; Lawler, 2005, 2014; Loveday, 2014a; Plummer, 2000; Reay, 2005; Skeggs, 2004a; Skeggs and Loveday, 2012; Tyler, 2008, 2013; Walkerdine, 1997; Walkerdine et al., 2001).

Not surprisingly given his interest in the role of education in reproducing social advantage (Bourdieu and Passeron, 1990), Bourdieu’s concepts have been widely taken up in the sociology of education.^4^ While HE is imagined as a field that has the potential to confer value through the accrual of different ‘forms of capital’ (Bourdieu, 1986), participation does not necessarily guarantee legitimacy (Loveday, 2014b; Quinn et al., 2005). Perceptions of ‘academic culture’ can determine the type of institution students choose to attend (Read et al., 2003), and ‘fitting in’ (Reay et al., 2010) can be a fraught process once students arrive at university. Existing studies on working-class participation in HEIs have explored different strategies and responses of these students to the predominantly middle-class field of the university (see e.g. Granfield, 1991 on disidentification; Reay et al., 2010 on reflexivity; Abrahams and Ingram, 2013 on the ‘chameleon habitus’).

However, in this article I focus on the relations *between classed actors* in the field of HE, where HE is understood as a ‘field of struggles’ (Bourdieu, 1990b) in which participants must compete for valuable forms of capital, ‘symbolic mastery’ (Bourdieu, 1986) and ‘person-value’ (Skeggs, 2010a, 2011). Bathmaker et al. (2013) explore emerging ‘processes of capital acquisition’ (p. 727) in HE in the form of extra-curricular activities and internships. They note that although access to HE might have expanded, ‘advantage is maintained through a shift in the rules of the game’ (p. 741), which favour some participants over others. The ability of actors to accrue capital – and ultimately ‘person-value’ (Skeggs, 2011, 2010a) – is contingent; only those whose embodied ‘dispositions’ (Bourdieu, 1990b: 190) are congruent with the field have a ‘feel for the game’ (Bourdieu, 1990b) and so are accepted as legitimate. Existing literature by academics from working-class backgrounds (see e.g. Mahony and Zmoroczek, 1997) suggests that even those who ostensibly appear to have accrued a large volume of capital by virtue of their professional positions may still experience considerable ambivalence about the roles they occupy in the field, and may also have their positions called into question by others (Hey, 2014). What role might ‘devices of distinction and differentiation’ (Skeggs and Loveday, 2012) play in foreclosing attempts to accrue ‘person-value’ (Skeggs, 2010a, 2011)?

I look at judgement here as one such device, which has the capacity to generate shame. The importance of early work foregrounding the significance of emotion to social class (Rubin, 1973; Sennett and Cobb, 1972) arguably took some time to be acknowledged, yet there is now an emerging literature on class and the embodiment of emotion and affect (Allen, 2014; Charlesworth, 2000; Dicks, 2008; Hey, 2014; Kirk, 2007; Lawler, 2005; Lucey et al., 2003; Reay, 2005; Skeggs, 2010b; Skeggs and Loveday, 2012; Tyler, 2008, 2013; Walkerdine, 2010; Walkerdine and Jiminez, 2012; Walkerdine et al., 2001). The focus of this article is the lived experience of shame for working-class participants in HE, yet how should shame be theorised? I have chosen to work with Wetherell’s (2012, 2014) concept of ‘affective practice’ here for two reasons. First, I want to think about how ‘[p]eople both actively practice and thus are agentic in that limited sense, but [how] they are also constituted as they practice and through their histories of past practice’ (Wetherell, 2014: 234). One of the strengths of drawing on this notion of ‘practice’ (see also Bourdieu, 1977, 1990a) is to acknowledge the way in which individuals might be constrained in their ability to act, yet without completely removing the possibility for agency, or indeed resistance. Second, while there is some debate as to whether emotion and affect should be distinguished (Greco and Stenner, 2008; Probyn, 2005), emotions have the tendency to be recognised as properties of the person, rather than as the result of social practice. As Probyn phrases it, this is the difference between ‘*being* affected and *having* an emotion’ (2005: 20; emphasis in original). By working here with the concept of ‘affective practice’, I want to illuminate how it is that shame becomes misrecognised as a classed and gendered property of individuals, rather than a symptom of inequality. How is it that a problem of society can so easily be turned into a deficiency of the self?

Below I summarise the empirical context of the article. I then go on to analyse two examples of classed embodiment in some depth: first, I foreground the example of accent in order to think through how moral judgment as an ‘affective practice’ precipitates shame, constructing working-class people as ‘deficient’; second, I explore two of my participants’ experiences of pregnancy in order to analyse how ‘affective practice’ is both classed and gendered. I conclude by considering what is at stake in the classed, lived experience of shame in English HEIs.

## Researching the Lived Experience of Class and ‘Affective Practice’

The concept of ‘lived experience’ underscores the stories presented in this article and has been informed by what McNay terms as Bourdieu’s ‘social phenomenology’:

[I]t provides a way of placing experience at the centre of social analysis without attributing to it some kind of apodictic or essential status. The idea of phenomenology as a relational rather than an ontological style of enquiry avoids the problem of the reification of ‘experience’ that hampers many kinds of interpretive analysis. (McNay, 2004: 184)

In this sense, I focus on the ‘lived experience’ of the participants in this research not to take recourse to the ‘evidence of experience’ (Scott, 1991; see also McNay, 2004), but to think through the social production of experience and, in particular, how relations between actors in the field of HE are constructed by social and historical ‘categories of representation’ (McNay, 2004: 179) that have unevenly attributed ‘person-value’ (Skeggs, 2010a, 2011).

The article is based on research conducted as part of a wider project, which sought to explore how working-class identities are experienced subjectively in English HEIs by thinking through the processes which might enable or constrain class-based identification in this particular field. All of the participants interviewed described themselves either as ‘working class’ or as coming from ‘working-class backgrounds’ and all were working and/or studying in English HEIs at the time of the research.

I had previously been employed by Open Book – a grass-roots Widening Participation project currently operating in four English HEIs, which supports students from socially excluded backgrounds. My professional involvement with Open Book sparked my initial interest in working-class experiences of HE. I had good existing relationships with staff at Open Book, meaning that eight colleagues volunteered to take part in the research. I subsequently took the decision to recruit other types of participants in order to widen the scope of the project. I recruited 11 academic staff and seven students, including a postgraduate student who was also working as a lecturer. In total, there were 14 female participants and 12 male, all of whom were from arts, humanities, or social science disciplines, and ranged in age from their early twenties to mid-fifties. While ‘race’ undoubtedly intersects with class and gender in mediating experiences of HE (see e.g. Mirza, 2008), all of the participants discussed here are White British, so it has not been possible to draw comparisons across the experiences of different ethnic groups. Recruitment involved using a mixture of existing contacts and networks, snowball sampling and, in the case of the students, an email circulated to academic departments advertising for participants from ‘working-class backgrounds’.

One of the most important ethical considerations of the research has been to protect the anonymity of participants who are anxious not to be recognisable in any way. Biographical details are given where possible to situate the participants’ narratives, but providing more precise information would in some instances compromise the anonymity and confidentiality of participants. A further consideration has been the need to think reflexively about my role as an ‘insider researcher’. As a sociologist working in an English HEI, I am emplaced within the same field as my participants. Prior to the commencement of the research, some of the project’s participants were known to me personally and professionally; negotiating existing relationships can be challenging (Owton and Allen-Collinson, 2014), but also beneficial (Taylor, 2011). For example, when asked if she had spoken to me ‘differently’ because of our existing relationship, Lisa – a postgraduate student aged in her thirties – responded that our previous conversations about class had ‘opened a way to talk with you candidly’ (see also Mercer, 2007). In this sense, it is important to consider ‘what the data are telling me that they might not tell someone else’ (Srivastava and Hopwood, 2009: 81).

The primary research method was the use of narrative-style interviews, which began by asking participants to tell the stories of their educational trajectories. While the generalisability of narrative accounts is contested, in his defence of case study research Flyvbjerg (2007: 395) claims that ‘formal generalisation is overvalued ... whereas “the force of example” is underestimated’. An inductive approach to data analysis was taken; interview transcripts were coded thematically and comparisons were made between cases. One of the key themes to emerge from the data analysis was the visceral experience of shame. However, there is some difficulty in adequately capturing the affective nature of experience whilst using a predominantly interview-based methodology since, as Walkerdine (2010: 92) notes, the interview ‘does tend to be very language based’. Yet some of the interviews conducted were deeply affective, and this was particularly exemplified in my interviews with Lisa and with Joe (the Open Book Coordinator, who is aged in his forties). Lisa became unexpectedly quite upset as she spoke and recounted her initial experiences of HE as a younger woman; at one point she appeared to be on the verge of tears. As she later explained:

[B]ecause we knew each other ... I thought I could just rattle off a few facts about myself and help you out but I was very surprised to find myself getting so emotional – it’s still so under the surface and suddenly I felt very strange – exposed and embarrassed that I was suddenly emotional and so angry.

Asking Lisa to recount her educational story brought to the ‘surface’ a number of painful ‘ugly feelings’ (Ngai, 2005). My interview with Joe was also affectively charged, but in a different sense. He became increasingly animated throughout our discussion, his voice raising. Much of his anger was directed towards contemporary discourses that represent the working classes as: ‘objects of sympathy at best and fucking scorn and derision at worst’.

I raise these incidences not merely as a means of reflecting on the methodological limits of the interview, but because they seem to point to the complex operation of social class at the affective level. As a researcher, I have not been privy to most of the interactions or events described to me by the participants. However, I believe it is a mistake to imagine that affect is only generated in the moment of a particular encounter or experience; the process of recollection is in itself a type of ‘affective practice’ (Wetherell, 2012, 2014) and part of the performative capacity of shame seems to be its ability to make itself felt – sometimes unexpectedly – even years after a specific experience; in Lisa’s interview for example, shame is viscerally re-lived. Yet in his outrage at the negative representation of the working classes, Joe conversely seeks to challenge discourses of devaluation. It is these contingencies of ‘affective practice’ that I explore further below.

## ‘She Can’t Even Speak Properly’

Moral judgements cast aspersions on who or what is valuable; to be positioned as worthless may have a number of external effects (structural, cultural or economic), but this positioning also often appears to be ‘internalized’ (Sayer, 2005: 153) or embodied by those who are deemed to be without worth. My interest in shame relates to the way in which certain actors are framed as ‘valueless’ (Skeggs and Loveday, 2012); in particular, contemporary representations of the working classes have been shown to be overwhelmingly negative (Skeggs, 2004a; Skeggs and Loveday, 2012; Skeggs and Wood, 2012; Tyler, 2008, 2013), as Joe articulates above. I want to suggest that ‘shame’ is particularly pertinent to the cultural analysis of class in that it often masquerades as a naturalised property of the self, obscuring the crucial role of *evaluation* within the ‘moral economy’ (Skeggs, 2009), which attributes value to some at the expense of others. This may help to explain how it is that working-class people may come to believe this story of inadequacy themselves.

In this sense, shame is not merely a residual effect of classed relationships; shame is part of the *practice* that feeds back into unequal relations, *shaping* perceptions and action and, ultimately, helping to reinforce such inequity. As Skeggs (2010a: 49) notes:

… affect is only significant if it is attached to ideas that matter: expressions of ‘just-talk’ and ‘ugly feelings’ make explicit the way value is circulating and attaching in unjust ways in the dominant symbolic. But they also work out … what is just, who and what is ‘worth it’ and in so doing, generate a composite of person-value … or worthlessness.

One of the reasons for conceptualising shame as the product of ‘affective practice’ (Wetherell, 2012, 2014) is in order to think relationally, whilst avoiding imagining that shame permanently resides within bodies (even if this is how they are sometimes felt). Anthony – an Open Book employee aged in his thirties – described how the low expectations that he associated with his class background ‘seep into you’. I have found this concept of ‘seepage’ to be productive in thinking about the relationality implicated in ‘affective practice’ and the ‘symbiotic relationship between feeling shame and being shamed’ (Chase and Walker, 2012: 743). How does shame ‘seep’ into some bodies more effectively than others?

In order to think through how judgement works as an ‘affective practice’ that facilitates this ‘seepage’ of shame, I turn now to the example of accent as a particular expression of classed embodiment. While I did not specifically ask my participants about accent, it was frequently cited as significant by all of the types of participant whom I interviewed, from undergraduate student to professor. As Dick explains, when he has to speak in public at the elite university where he works as a professor in criminology: ‘I’m well aware of my accent in a way that I’ve never been before in my life.’ In the UK, accents vary enormously across different regions, but are also significant markers of social class position (Hey, 1997) and so their evaluation is often far from neutral (Addison and Mountford, 2015).

Plummer (2000: 43) argues that ‘the all-pervasive social pathology model – inadequate working-class homes, language and culture – is still with us’. In sociolinguistics, there has been a long-standing debate about the relationship between language use, class and ‘cultural deficiency’, exemplified by Bernstein’s (1971) work on the so-called ‘restricted’ linguistic ‘codes’ of the working classes.^5^ Bourdieu (1991: 411) notes:

What is rare, then, is not the capacity to speak ... but rather the competence necessary in order to speak the legitimate language which, depending on social inheritance, re-translates social distinctions into the specifically symbolic logic of differential deviations, or, in short, distinction.

The ‘linguistic habitus’ (Bourdieu, 1991) of a speaker arguably has the potential either to depreciate value for those who speak ‘out of place’, or conversely to accrue ‘symbolic mastery’ (Bourdieu, 1986) for those whose speech is congruent with the expectations of the field, a finding also supported by Addison and Mountford (2015) in their research on social class, accent and value in HE.

During my time working at Open Book, a student – who spoke with a regional accent – recounted how one of his lecturers had told him that his accent was ‘disgusting’. Ahmed (2005: 93) asserts that ‘to name something as disgusting … is performative’. The naming of an accent as ‘disgusting’ marks it out in this way, and simultaneously reinforces the distinction of the person who names it as such (see Lawler, 2005). However, this type of ‘naming’ also works in tandem with the extra-linguistic, particularly ‘affective practice’ (Wetherell, 2012, 2014). What has been interesting to me throughout the wider project is how the ‘affective practice’ implicated in ‘speaking class’ differs. While all of the participants who mentioned the role of accent are at least implicitly aware of its shaming potential, this potentiality appears to be experienced in quite contingent ways. For example, Joe remains defiant in the face of negative evaluation of his Cockney accent: ‘I will never change the way I speak for anyone.’ His defiance suggests both a refusal to ‘submit’ to the ‘demands of the field’ (Skeggs, 2004b: 29, following Bourdieu) and a rejection of the legitimacy of that field’s dominant values (see also Loveday, 2014b; Skeggs, 2011; Skeggs and Loveday, 2012). Conversely, other participants felt acutely shamed by their accents, such as in the case of Lisa who recounts actively trying to adapt her regional accent when she first arrived at university to the point where ‘my voice wasn’t my own’.

Various types of occasion and interaction were described by the participants when their speech became the locus of ‘affective practice’ (Wetherell, 2012, 2014), and evaluation appears to be key to the precipitation of shame. Two participants originally from the same area of England – the Midlands – describe how their accents have been positioned as making them sound ‘stupid’. Ruth, now a professor, recounts an incident experienced as an undergraduate student:

And one of my seminars was a seminar on language ... and a girl with ... such beautiful received pronunciation of the like I’d never heard before said she couldn’t stand the local accent and she’d been to the shop and this is how they spoke and how terrible it was and how stupid people sounded. And I was just paralysed because I thought, ‘that’s me’.

Hannah – an undergraduate student in her early twenties – tells a similar story to that of Ruth:

I have to work hard to speak ... And people ... start talking to you like you’re dumb when you’ve got a bit of an accent ... it lowers their opinion of you ... Certain words sound kind of stupid. Everyone starts taking the piss [teasing] a bit.

While Ruth is ‘paralysed’ as she recognises her peer’s evaluation of the local accent, Hannah gestures towards the performative capacity of this type of assessment; while she explains that people assume she is ‘dumb’ and sometimes tease her, the ‘affective practice’ (Wetherell, 2012, 2014) of these judgements also leads to self-evaluation. She continues on to imagine how it must be that others appraise her accent when she comments: ‘they’re like, “she can’t even talk properly”’. Fiona is an Open Book employee, with a master’s degree in History of Art, aged in her forties and raised in southeast London. In our interview she feels that she has been perceived as ‘aggressive’ within the university environment, because of her Cockney accent. She discusses the result of this type of ‘feedback’:

for me it’s about trying to present myself in a way that’s the most perfect way I could present myself ... so I’ve been in a constant editorial process with myself.

Self-regulation here may foreclose the potential for shame; in order to avoid the shame associated with being marked out as ‘aggressive’, Fiona engages in an ‘editorial process’ to present an ‘acceptable’ version of herself to others, a process similar to that described by Hannah above, who has ‘to work hard’ when she speaks.

The imagined moral evaluation of one’s self-worth – or the ‘fear of being summoned before some hidden bar of judgement’ (Sennett and Cobb, 1972: 33), appears to have as powerful an effect as the real gaze of a judgemental Other. It may also act as a type of self-regulatory force, such as in the cases of Hannah and Fiona above. Chase and Walker (2012: 740) elaborate on the ‘co-construction’ of shame as:

… combining an internal judgement of one’s own inabilities; an anticipated assessment of how one will be judged by others; and the actual verbal or symbolic gestures of others who consider, or are deemed to consider, themselves to be socially and/or morally superior to the person sensing shame.

Calculations of ‘person-value’ (Skeggs, 2010a, 2011) are fundamental to this ‘co-construction’ of shame. Wetherell’s (2012, 2014) notion of ‘affective *practice*’ is helpful here as it allows for the consideration of what affects such as shame *do* (Ahmed, 2004: 4). It is not surprising that to be told that your accent is ‘disgusting’, or that you sound ‘stupid’, is potentially shaming. Yet the ways in which the participants react to judgement are significant. Below, I want to consider the stories of Tina and Lisa, in order to think through further the significant role that gender plays in the ‘affective practice’ of judgement and the corresponding experience of classed, embodied shame.

## ‘A Symbol of Shame’

In our interview, Lisa contrasts her own class identification to that of her partner. In particular, she notes that he ‘wants to hold on to that working-class background’, yet for Lisa, this is a potentially fraught strategy as class ‘pride’ carries with it the potential for shaming, which Lisa articulates when she worries that ‘he’s showing me up’. This response simultaneously makes her feel ‘ashamed’:

I’m talking about how I would feel if somebody judged me like that and … I’d be mortified.

In this sense, disidentification is a response to the shaming potential of class that accompanies devaluation (see Skeggs, 1997). As Sayer (2005: 160) notes: ‘the desire to be respectable and recognized as such is a shame response dependent on some degree of positive feeling towards what is lacked’. The ‘hidden bar of judgment’ (Sennett and Cobb, 1972: 33) that Lisa invokes is a strategy for avoiding the shame that she feels will be generated if she is exposed, by association, as being working class. While Lisa – as a social science student – is well aware of the social processes that determine ‘person-value’ (Skeggs, 2010a, 2011), the ‘affective practice’ (Wetherell, 2012, 2014) of judgement is still a real source of anxiety for her and she fears the negative valuation associated with being positioned as a working-class woman. I have previously explored male strategies of working-class identification, which refute the legitimacy of the ‘dominant symbolic’ (Loveday, 2014a; see also Skeggs and Loveday, 2012). What is it, then, about being a working-class woman that may specifically engender the potential for shame? While the vast majority of the male participants in my research did not fear being recognised as ‘working class’ – and indeed some participants, such as Joe and Neil (Open Book) and Steve (a PhD student/lecturer) describe actively attempting to be recognised as such – a greater proportion of the female participants expressed ambivalent feelings as to their class position. This, in turn, feeds into the ‘affective practice’ (Wetherell, 2012, 2014) of class positioning. While participants were certainly aware that they were sometimes judged negatively, this was no guarantee that shame would be ‘internalized’ (Sayer, 2005: 153); it seemed that the women in my research were more likely to have experienced feelings of shame associated with their class positioning and would then accordingly judge themselves more harshly. The intersection of gender with class appears to shape the practices involved in positive working-class identification (see also Skeggs, 1997).

It was interesting to me that two participants provided accounts of their pregnancies in the interviews I conducted, and these were not only rooted in gender, but also in their classed positioning. I refer to these two cases not in an attempt to generalise the specificity of these experiences to all working-class women in HE, but in order to explore how these participants make sense of their positioning in this particular field. I want to think through the story of Tina (an academic) in order to consider the intersection of class and gender in the ‘affective practice’ (Wetherell, 2012, 2014) of judgement. A significant part of Tina’s narrative was an unexpected pregnancy, which occurred during her PhD. Tina describes informing her supervisor (whom she describes as ‘posh’):

She was furious about me being pregnant, absolutely furious. And she said: ‘You’re the reason women ..., women like you shouldn’t get funding’.

Although what the supervisor means by the phrase ‘*women like you*’ is not made explicit, this statement is profoundly judgemental: implicit in this response is an idea about the nature of a deserving, or worthy, woman. Tina and women ‘like her’ are accordingly shamed and de-valued. Tina understands this scenario in terms of her class positioning, so that her class is projected onto this interaction. Tina describes how ‘any ability to pass’ – that is, to be (mis)recognised as middle class in the university in order to engender respectability – was ‘blown out the window’. The appearance of her pregnancy made Tina feel as if her body had been both visually classed and gendered, an exposure which she describes as ‘*a symbol of shame*’ within the university. She describes this experience as ‘being put back into your body all the time’. Walkerdine et al. (2001: 187) state:

The pressures of the fecund female body present a problematic path through education and life, whatever the class position. What is important is how the fecundity is regulated and lived. For middle-class young women it is their inscription as the bourgeois subject that counterposes fecundity in a way that simply does not allow the possibility of pregnancy.

Becoming pregnant as a PhD student is for Tina a visible and embodied display of her class positioning. Tina is marked out by her ‘fecundity’ (Ussher, 2006; Walkerdine et al., 2001), as are all *women like her*. The pregnancy prevents Tina from completing her PhD, but despite this she subsequently takes on an academic role involving a heavy load of teaching and administration. However, her failure to have completed the PhD impacts upon her negotiation of the workplace, a negotiation which Tina experiences as classed:

[Not finishing the PhD] became this incredible source of shame for me, that was tied up with class and, partly my shame at feeling ashamed. It became this, kind of, circle of shame, I felt ashamed that I didn’t have a PhD, so, on all the minutes for the committees I’d be ‘Ms’ and everyone else would be ‘Dr’ and I thought that reinforced this narrative round me that I was the one who did all the teaching. I was the one, people said to me: ‘Oh, you’re really fantastic at admin.’ But it was like I was repositioned, as a body, with the secretaries ... I had the same accent as all the secretaries ... I was literally aligned with their bodies and I was seen as a sort of blip.

Lacking a PhD when she begins her career impacts her status, but Tina also perceives her body as being classed through its conceptual ‘alignment’ with the secretaries. Returning to the significance of accent in ‘affective practice’ (Wetherell, 2012, 2014) once more, Tina speaks with a local accent unlike the other academics in her department. This, in a sense, ‘emplaces’ her body, whilst ‘othering’ her and making her feel like a ‘blip’. As Tina is ‘named’ as a type of glorified administrator – ‘Oh, you’re really fantastic at admin’, this becomes performative and she experiences her shame as being tied up with embodied classed and gendered markers in this particular field.

Lisa was the second participant to discuss her pregnancy, which also took place during the course of her PhD. She explains experiencing a feeling of discomfort on the visibility of her pregnant body:

I can say that in the academic environment I felt intensely uncomfortable when my body began to appear obviously pregnant and why was this? It was odd, I felt something akin to guilt.

Munt (2008: 8) contrasts guilt and shame by noting that ‘in the former one *knows* one has committed a wrong (guilt), and because of it, one has entered a state of disgrace (shame)’. Yet why does Lisa have this affective reaction? She speculates:

I think this was because of the feelings of entitlement – or lack of it – that relate to my journey from a working-classed background into this academic environment.

Entitlement relates to legitimacy here: who has the capacity to accrue valuable forms of capital (Bourdieu, 1986)? A congruence between the ‘dispositions’ of actors and the ‘demands of the field’ (Skeggs, 2004b) determines who is seen to have ‘person-value’ (Skeggs, 2010a, 2011) and this is so often misrecognised as ‘natural’, and so legitimate (see also Reay et al., 2005: 67, following Bourdieu). If the working-class body is potentially ‘disruptive’, then the embodiment of ‘feminine excess’ and ‘unruly fecundity’ (Ussher, 2006: 161) seems to be doubly disturbing in this environment.

I have been arguing that certain forms of embodiment (such as accented speech or pregnancy) challenge established boundaries of ‘appropriateness’, so that the mere presence of a body creates a feeling of disorder within a specific social field. Lisa notes how she felt as if her body became ‘disruptive’ while she was pregnant:

when I was in academic environments such as campus, a day conference and the academic groups I’m a member of, I felt so awkward – disruptive somehow ... why did I feel this way? Overall, people were overwhelmingly positive and warm but my embodied feeling ... was one of apology.

Why should the experience of being pregnant in academic environments have generated for both of these women a range of ambivalent emotions? I want to argue that this is tied up with how they understand their class positioning in HE, as well as social conceptions of the female body. While it is true that many women have the ‘potentiality for birth’ (Tyler, 2000: 292), the young, female working-class body is viewed as particularly excessive and overtly sexual; pregnancy becomes the ultimate symbol of uncontainable ‘feminine excess’ (Ussher, 2006: 161), but in the context of this particular field, also a symbol of classed excess and ‘abjection’ (Ussher, 2006: 161). For Lisa and Tina, their pregnant bodies visibly disrupted the dominant norms of the field and this disruption is experienced as shameful. I want to conclude below by arguing that the ‘affective practice’ (Wetherell, 2012, 2014) described in this article is not only the effect of inequality in this field, but is a mechanism that feeds back into classed relationships, variously shoring up notions of (il)legitimacy by contributing to processes of valuation.

## Conclusion

This article has explored the role of shame in mediating the experience of working-class staff and students in English HEIs through the use of two different examples of embodiment: accent and pregnancy. The findings presented here are based on a small-scale empirical study and so are not generalisable. However, the stories presented point to the different ways that the participants have made sense of their classed and gendered identities in this field, and how shame has constrained and affected action. Building on Bourdieu’s concepts of habitus, field and capital, I have sought to present a feminist reading of embodiment, practice and value. I have attempted to demonstrate that judgement – as part of a nexus of what Wetherell (2012, 2014) refers to as ‘affective practice’ – deflects analysis from the root causes of devaluation in a highly classed field, such as English HE, by shaming certain actors. ‘To be found out’ – that is, to be exposed as working class through certain classified forms of taste or behaviour (Bourdieu, 1984) – equated to being ‘humiliated’ for Lisa as a younger woman, and she understands this shame as ‘stick[ing] to your bones’. I have sought to highlight how the ‘stickiness’ (Ahmed, 2010; Munt, 2008) of shame not only has an enduring bodily presence as it acquired through practice over time, but might also act to shape the impression that working-class people have of themselves, as well as the types of relationships that they are able to form with people and environments.

Through an exploration of classed relationships in HE, I have sought to explore the conditions under which judgement has precipitated shame for the participants discussed in this article and how, over time, ‘affective practice’ (Wetherell, 2012, 2014) becomes performative. In this sense, shame has two sociologically significant roles. First, shame *feeds back* into legitimated schemas of valuation, reinforcing the ‘deficit’ view of working-class culture and identities (Plummer, 2000). Second, the participants here have described how shame is experienced by them as embodied, that is, how it becomes a part of the habitus acquired through ‘affective practice’. In this sense, shame naturalises person-deficit. By examining the ‘affective practice’ (Wetherell, 2012, 2014) of judgement, I have argued that a focus on the lived experience of shame helps to explain how deficiency becomes embodied, naturalising privilege and obscuring the ‘moral economy’ (Skeggs, 2009). In this context, shame works on bodies in their relationality *to other bodies* as actors ‘struggle’ over valuable forms of capital (Bourdieu, 1986) at stake in the educational field. It is the fundamentally relational nature of social class – as it intersects with other social processes, such as gender – that makes class itself into an ‘affective practice’.

## Notes

1 Widening Participation departments, funded by the Higher Education Council for England, have been established within English HE institutions to widen access to university for under-represented groups.

2 OFFA (2014: 2) notes that ‘the most advantaged 20 per cent of young people’ were 6.3 times more likely to go to HEIs with the highest entry requirements than ‘the most disadvantaged 40 per cent’ in 2011/12.

3. See, for example, the collection of chapters in Adkins and Skeggs (2004).

4. See Reay (2004) on the use of ‘habitus’ in educational research.

5. See Keddie (1973); Labov (1979); Rosen (1972) for critical analysis of the ‘deficit’ perspective; see Jones (2013) for an overview of the debate.
